# The Role of Mitochondria-Derived Reactive Oxygen Species in Hyperthermia-Induced Platelet Apoptosis

**DOI:** 10.1371/journal.pone.0075044

**Published:** 2013-09-04

**Authors:** Zhicheng Wang, Feng Cai, Xiaoyu Chen, Meihong Luo, Lingling Hu, Yuan Lu

**Affiliations:** 1 Department of Laboratory Medicine, Huashan Hospital, Shanghai Medical College, Fudan University, Shanghai, China; 2 Department of Laboratory Medicine, Shanghai Hospital of Traditional Chinese Medicine, Shanghai University of Traditional Chinese Medicine, Shanghai, China; 3 Department of Histology and Embryology, Anhui Medical University, Hefei, China; University of Pittsburgh School of Medicine, United States of America

## Abstract

A combination of hyperthermia with radiotherapy and chemotherapy for various solid tumors has been practiced clinically. However, hyperthermic therapy has side effects, such as thrombocytopenia. Up to now, the pathogenesis of hyperthermia-induced thrombocytopenia remains unclear. Previous studies have shown that hyperthermia induces platelet apoptosis. However, the signaling pathways and molecular mechanisms involved in hyperthermia-induced platelet apoptosis have not been determined. Here we show that hyperthermia induced intracellular reactive oxygen species (ROS) production and mitochondrial ROS generation in a time-dependent manner in platelets. The mitochondria-targeted ROS scavenger Mito-TEMPO blocked intracellular ROS and mitochondrial ROS generation. By contrast, inhibitors of reduced nicotinamide adenine dinucleotide phosphate (NADPH) oxidase, nitric oxide synthase, cyclooxygenase and lipoxygenase did not. Furthermore, Mito-TEMPO inhibited hyperthermia-induced malonyldialdehyde production and cardiolipin peroxidation. We also showed that hyperthermia-triggered platelet apoptosis was inhibited by Mito-TEMPO. Furthermore, Mito-TEMPO ameliorated hyperthermia-impaired platelet aggregation and adhesion function. Lastly, hyperthermia decreased platelet manganese superoxide dismutase (MnSOD) protein levels and enzyme activity. These data indicate that mitochondrial ROS play a pivotal role in hyperthermia-induced platelet apoptosis, and decreased of MnSOD activity might, at least partially account for the enhanced ROS levels in hyperthermia-treated platelets. Therefore, determining the role of mitochondrial ROS as contributory factors in platelet apoptosis, is critical in providing a rational design of novel drugs aimed at targeting mitochondrial ROS. Such therapeutic approaches would have potential clinical utility in platelet-associated disorders involving oxidative damage.

## Introduction

A combination of hyperthermia with radiotherapy and chemotherapy has been clinically applied for various solid tumors [1–3]. Thus, the biological effects of hyperthermia have been extensively studied. The induction of apoptosis has been proposed as a mechanism for hyperthermia-induced cell killing [2,3]. However, hyperthermia therapy has some side effects, such as thrombocytopenia [4,5]. Up to now, the pathogenesis of hyperthermia-induced thrombocytopenia remains unclear. We previously studied hyperthermia-induced platelet apoptosis [6], and our observations suggested that hyperthermia-induced platelet apoptosis might contribute to hyperthermia-triggered thrombocytopenia. However, the signaling pathways and molecular mechanisms responsible for hyperthermia-induced platelet apoptosis have not been well studied.

Hyperthermia induces reactive oxygen species (ROS) in various cell types, wherein ROS play an important role as intracellular mediators of hyperthermia-induced apoptosis [7,8]. ROS, including superoxide, hydrogen peroxide, and hydroxyl radicals, might also play pivotal roles in both physiological and pathological processes, including cell adhesion, growth, differentiation, viability and apoptosis [7–14]. Several potential sources of ROS have been suggested, and these include mitochondria, reduced nicotinamide adenine dinucleotide phosphate (NADPH) oxidase, xanthine oxidase and uncoupled nitric oxide synthase [15].

Mitochondria are a major source of ROS in most cells [11]. The formation of ROS occurs when unpaired electrons escape the electron transport chain and react with molecular oxygen, generating superoxide [11]. Complexes I and III of the electron transport chain are the major potential loci for superoxide generation [15]. Quinlan et al. reported that mitochondrial complex II can generate ROS at high rates in both the forward and reverse reactions [16]. ROS degradation is performed by endogenous enzymatic antioxidants such as superoxide dismutase (SOD), glutathione peroxidase (GPx), catalase and non-enzymatic antioxidants such as glutathione, ascorbic acid, α-tocopherol, carotenoids or flavonoids [11,14,17].

Under physiological conditions, ROS are maintained at proper levels by a balance between its synthesis and its elimination. An increase in ROS generation, a decrease in antioxidant capacity, or a combination both will lead to oxidative stress [18]. Recently, several studies have identified NADPH oxidase-derived ROS as key intermediates in hyperthermia-induced apoptosis [19,20]. By contrast, few studies have focused on mitochondria as a source of ROS in hyperthermia-induced apoptosis. In recent years, mitochondria-targeted ROS antagonists and mitochondrial ROS detection probes have been developed. Thus, with the advent of such tools, the importance of mitochondrial ROS in cell signaling, proliferation, differentiation and apoptosis gradually attracted much attention [11–15,21–25]. Dikalova et al. reported that mitochondrial ROS is important in the development of hypertension, and that mitochondria-targeted antioxidant Mito-TEMPO decreased mitochondrial ROS, inhibited total cellular ROS, and restored the levels of bioavailable nitric oxide [21].

Mitochondrial ROS might play a key role in the failure of pancreatic β-cells in the pathogenesis of type 2 diabetes [22]. Mitochondria-targeted antioxidants protect pancreatic β-cells against oxidative stress and improve insulin secretion in glucotoxicity and glucolipotoxicity [22]. Excess generation of ROS in the mitochondria acts as mediators of the apoptosis signal transduction pathways. Vela et al. reported that mitochondrial ROS plays an important role in iminophosphorane-organogold (III) complexe-induced cell death [23]. Loor et al. reported that during ischemia mitochondrial ROS triggers mitochondrial permeability transition pore (MPTP) activation, mitochondrial depolarization, and cell death during reperfusion [24]. Venkataraman et al. reported that PC-3 cells that overexpress manganese superoxide dismutase (MnSOD) had decreased synthesis of ROS, less lipid peroxidation, and greater cell survival as compared with wild-type PC-3 cells subjected to hyperthermia [25]. This observation suggested that mitochondria-derived superoxide anions play pivotal roles in the cytotoxicity that is associated with hyperthermia.

Although oxidant stress and apoptosis have both been implicated in hyperthermia-treated cell death, the relationship between these processes is not clearly established in platelets. The present study explored whether ROS play a role in hyperthermia-induced platelet apoptosis. We have used various pharmacological inhibitors to explore the sources of ROS in hyperthermia-treated platelets. We demonstrate the mechanisms involved in the apoptosis of hyperthermia-treated platelets.

## Materials and Methods

### Reagents and Antibodies

Trans-epoxysuccinyl-L-leucylamido(4-guanidino) butane (E64), GM6001 were obtained from Calbiochem (San Diego, California). Anti-cleaved p17 fragment of caspase-3 antibody was obtained from Millipore (Billerica, Massachusetts). Mito-TEMPO was obtained from Enzo Life Sciences (Plymouth Meeting, Pennsylvania). Calcium ionophore A23187, adenosine diphosphate (ADP), tetramethylrhodamine ethyl ester (TMRE), thrombin, aprotinin, phenylmethylsulfonyl fluoride (PMSF), N-acetylcysteine (NAC), 5,8,11,14-eicosatetraynoic acid (ETYA), *N*ω-Nitro-L-arginine methyl ester hydrochloride (L-NAME), apocynin, diphenylene iodonium (DPI), 2’, 7’-dichlorofluorescin diacetate (DCFDA) were obtained from Sigma (St. Louis, Missouri). Monoclonal antibodies against Bax, cytochrome C, tubulin, cytochrome C oxidase subunit 1 (COX1), actin, manganese superoxide dismutase (MnSOD), phospholipid hydroperoxide glutathione peroxidase (GPx4) and anti-GPIbα N-terminal antibody (SZ-2), FITC-conjugated goat anti-mouse IgG, and HRP-conjugated goat anti-mouse IgG were obtained from Santa Cruz Biotechnology (Santa Cruz, California). FITC-conjugated annexin V was obtained from Bender Medsystem (Vienna, Austria). 10-N-nonyl acridine orange (NAO), MitoSOX^TM^ Red were obtained from Invitrogen/Molecular Probes (Eugene, OR). The malonyldialdehyde (MDA) assay kit was obtained from Beyotime Institute of Biotechnology (Beyotime, Nantong, China). Mitochondria isolation kit was obtained from Pierce (Rockford, IL, USA). Factor VIII-free human von Willebrand factor (VWF) was obtained from Hematologic Technologies, Inc. (Essex Junction, VT).

### Preparation of Platelet-Rich Plasma (PRP) and Washed Platelets

This study was approved by the Medical Ethical Committee of Huashan Hospital, Fudan University, under permit number MEC-HS (Hu) 2011-362. The ethics committee/institutional review board included Hejian Zou, Yong Gu, Yingyuan Zhang, Chuanzhen Lu, Weihu Fan, Dayou Wang, Jianhua Zhang, Zhongrui Lu, Quanxing Ni. All patients signed informed consent for the collection and use of his/her blood for this study. PRP and washed platelets were prepared as described previously [26]. Briefly, fresh blood from healthy volunteers was anti-coagulated with a 1/7 volume of acid-citratedextrose (ACD, 2.5% trisodium citrate, 2.0% D-glucose and 1.5% citric acid). Anti-coagulated blood was separated by centrifuging at 150 × *g* for 20 min at room temperature (RT), and the supernatant was PRP. PRP was centrifuged at 1300 × *g* for 10 min at RT, and platelet precipitate was washed twice with CGS buffer (123 mM NaCl, 33 mM D-glucose, 13 mM trisodium citrate, pH 6.5). The washed platelets were re-suspended in modified Tyrode’s buffer (MTB) (2.5 mM Hepes, 150 mM NaCl, 2.5 mM KCl, 12 mM NaHCO_3_, 5.5 mM D-glucose, pH 7.4) to a final cell density of 3 × 10^8^ /ml. In PRP experiments, fresh blood was anti-coagulated with 1/9 volume of 3.8% trisodium citrate, and centrifuged at 150 × *g* for 12 min at RT to obtain PRP. Next, washed platelets and PRP were incubated at RT for 1 hour (h) to recover to the resting state.

### Measurement of Total Cellular ROS and Mitochondrial ROS Levels

Total cellular ROS and mitochondrial ROS levels were examined using DCFDA and MitoSOX^TM^ Red, respectively, according to the manufacturer’s instructions. Briefly, washed platelets were loaded with DCFDA (10 µM) or MitoSOX^TM^ Red (5 µM) at 37°C for 20 min in the dark and washed three times with MTB. Pre-loaded platelets were incubated at RT, 37°C, 40°C or 42°C for 1, 2, or 3 h respectively. For the inhibition experiments, pre-loaded platelets were incubated with DPI (20 µM), apocynin (100 µM), NAC (2 mM), Mito-TEMPO (10 µM), L-NAME (100 µM), ETYA (50 µM), or solvent control at 37°C for 15min, and then treated at different temperatures for 3 h. Thrombin-treated and antimycin A-treated platelets were used as positive controls for total cellular ROS and mitochondrial ROS levels, respectively.

### Assessment of Cardiolipin Peroxidation

Cardiolipin peroxidation was determined by using 10-N-nonyl acridine orange (NAO), a highly specific fluorescent probe for cardiolipin. After the peroxidation of cardiolipin, NAO loses its affinity for peroxidized cardiolipin, resulting in a decreased fluorescent signal [27]. Washed platelets were incubated at RT, 37°C, 40°C or 42°C for 3 h, and then loaded with NAO at 5 µM and incubated for 30 min at 37°C. Next, the platelets were washed with MTB and analyzed by flow cytometry. For the inhibition experiments, platelets were pre-incubated with Mito-TEMPO (10 µM) or solvent control at 37°C for 15min and then incubated at different temperatures for 3 h.

### Measurement of Malonyldialdehyde (MDA) Levels

MDA is a product of lipid membrane oxidation and a marker of oxidative damage [28]. MDA levels were measured using an MDA assay kit according to the manufacturer’s protocol. Briefly, washed platelets were incubated at RT, 37°C, 40°C or 42°C for 3 h, treated with cell lysis buffer and centrifuged at 10000 × *g* for 15 min. The supernatants were incubated with thiobarbituric acid (TBA), and the absorbance of the supernatants was measured using a spectrophotometer at a wavelength of 535 nm. For the inhibition experiments, platelets were pre-incubated with Mito-TEMPO (10 µM) or solvent control at 37°C for 15min. and then incubated at different temperature for 3 h.

### Measurement of Mitochondrial Inner Transmembrane Potential (*Δ*Ψm)

Measurement of ΔΨm was determined using the potential-sensitive dye TMRE. Briefly, washed platelets were pre-incubated with Mito-TEMPO (10 µM) or solvent control at 37°C for 15 min, and then incubated at different temperatures for 3 h. TMRE was added to the treated platelets to a final concentration of 100 nM. Next, the samples were incubated in the dark at 37°C for 20 min, and analyzed by flow cytometry.

### Phosphatidylserine (PS) Externalization Assay

The PS externalization was determined according to a previously published procedure [29]. Briefly, washed platelets were pre-incubated with Mito-TEMPO (10 µM) or solvent control at 37°C for 15 min, and then incubated at different temperatures for 3 h. Annexin V binding buffer was mixed with treated platelets and FITC-annexin V at a ratio of 50:10:1 respectively. Samples were gently mixed and then analyzed by flow cytometry.

### Subcellular Fractionation

Platelet subcellular fractionation was performed using a commercial mitochondria isolation kit as described [30]. Briefly, washed platelets were incubated at RT, 37°C, 40°C, or 42°C for 3 h, and were then further suspended in mitochondria isolation buffer A and lysed with buffer B at 4°C. Samples were further mixed with buffer C followed by centrifugation. The supernatants derived from the platelet lysates were further centrifuged at 12000 × *g* for 20 min to yield the mitochondrial pellet and the mitochondria-free cytosolic fraction (supernatant). For inhibition experiments, platelets were pre-incubated with Mito-TEMPO (10 µM) or solvent control at 37°C for 15min, and then further incubated at 42°C for 3 h.

### Western Blot Analysis

After subcellular fractionation Bax and cytochrome C were detected by SDS-PAGE and Western blot using anti-Bax, and anti-cytochrome C antibody, and as described above. COX1 and tubulin were used as mitochondrial and supernatant internal controls, respectively. Expression of MnSOD, GPx4, and caspase-3 activation was assessed with platelet whole lysates. Washed platelets were incubated at RT, 37°C, 40°C or 42°C for 3 h and lysed with an equal volume of lysis buffer containing 0.1 mM E64, 1 mM PMSF and 1/100 aprotinin on ice for 30min. The samples were subjected to SDS-PAGE and Western blot analysis using anti-caspase-3, anti-MnSOD, anti-GPx4, respectively. Anti-actin antibody was used as an equal protein loading control. In the inhibition experiments, platelets were pre-incubated with Mito-TEMPO (10 µM) or solvent control at 37°C for 15 min, and incubated at 42°C for 3 h.

GPIbα ectodomain shedding was assessed with supernatants as described previously [6]. Briefly, washed platelets were incubated at RT, 37°C, 40°C or 42°C for 3 h. Samples were centrifuged at 4000 rpm for 5 min to harvest the supernatants. The samples were subjected to SDS-PAGE and Western blot analysis using anti-GPIbα N-terminal antibody SZ-2. For the inhibition experiments, platelets were pre-incubated with Mito-TEMPO (10 µM), GM6001 (100 µM), or solvent control at 37°C for 15 min, and then further incubated at 42°C for 3 h.

### Platelet Aggregation

Platelet aggregation was performed using a turbidometric platelet aggregometer. Briefly, PRP or washed platelets were pre-incubated with or without Mito-TEMPO (10 µM) and solvent at 37°C for 15 min, and then incubated at 42°C for 2 h. Platelet aggregation was induced by addition of ADP or thrombin at 37°C with a stirring speed of 1000 rpm.

### Platelet Adhesion Under Flow Conditions

Human von Willebrand factor (vWF) was diluted to 30 µg/ml with 0.1 M NaHCO_3_ (pH 8.3), and coated onto glass capillary tubes overnight in a humid environment at 4°C. The capillaries were washed with phosphate buffer saline (PBS), blocked with 5% bovine serum albumin (BSA) in PBS at 22°C for 2 h, and then mounted on to the stage of an inverted microscope as described previously [31]. Washed platelets, were pre-incubated at different temperature for 3 h, then perfused into the glass capillary by a syringe pump at a flow shear rate of 250 s^-1^ for 5 min, and then washed with MTB for 5 min. The number of adherent platelets was counted in 10 randomly selected fields of 0.25 mm^2^ and at randomly selected time points. For the inhibition experiments, platelets were pre-incubated without (control) or with Mito-TEMPO (10 µM) and solvent at 37°C for 15 min, and then incubated at 42°C for 3 h.

### Statistical Analysis

The experimental data were expressed as mean ± SEM. Each experiment was carried out at least three times. Statistical analysis for multiple group comparisons were performed by one-way analysis of variance (ANOVA), followed by post-hoc Dunnett’s test. A *P*-value of less than 0.05 was considered statistically significant.

## Results

### Mitochondria were the major sources of ROS in hyperthermia-treated platelets

**Figure 1:**
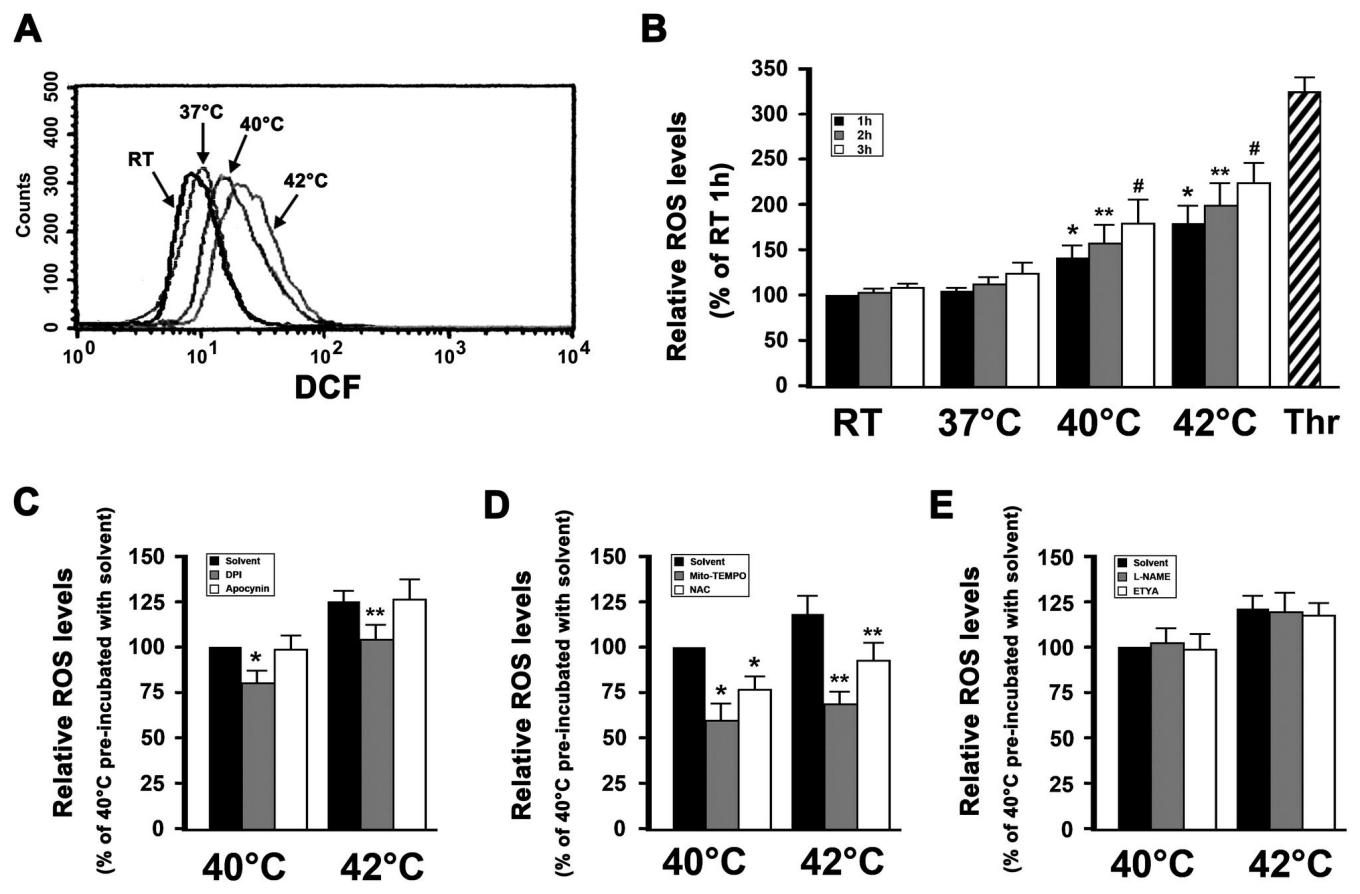
Mitochondria are the major sources of ROS in hyperthermia-treated platelets. (A and B) DCFDA-loaded platelets were incubated at the indicated temperatures for 3 h (A) or 1-3 h (B). As a positive control, loaded platelets were incubated with thrombin at 37°C for 30 min. Samples were then analyzed for intracellular ROS levels by flow cytometry. Representative flow cytometric histogram is shown (A). The relative ROS levels are expressed as a percentage of platelets, which were incubated at RT for 1 h (B). Percentage of RT 1h is presented as mean ± SEM from three independent experiments. *P<0.017 (after Bonferroni correction) as compared with RT 1 h, **P<0.017 (after Bonferroni correction) as compared with RT 2 h, #*P*<0.017 (after Bonferroni correction) as compared with RT 3h. (C–E) DCFDA-loaded platelets were pre-incubated with solvent control, DPI and apocynin (C), Mito-TEMPO and NAC (D), or L-NAME and ETYA (E) at 37 °C for 15 min, and then incubated for 3 h at 40°C or 42°C, and further analyzed by flow cytometry. The relative ROS levels are expressed as a percentage of platelets, which were pre-incubated with solvent control at 37°C for 15 min and then incubated at 40°C for 3 h. Percentage of 40°C loaded platelets pre-incubated with solvent control is presented as mean ± SEM from three independent experiments. *P<0.025 (after Bonferroni correction) as compared with solvent control at 40°C, **P<0.025 (after Bonferroni correction) as compared with solvent control at 42°C. Thrombin is labeled as Thr.

Hyperthermia induces ROS production in various cell-lines and in tumor tissues, wherein heat-induced oxidative stress appears to play a pivotal role in the induction of apoptotic cell death [7,8]. Our previous study has shown that hyperthermia induces platelet apoptosis [6]. In order to investigate whether hyperthermia augments intracellular ROS levels in platelets, we determined platelet ROS levels using DCFDA, which is a sensitive indicator of total cellular ROS. ROS production was significantly increased in platelets incubated at 42°C for 3 h (Figure 1A and 1B) and did so in a time-dependent manner and concordantly with increases in temperature. Therefore, in order to obtain detectable effects, a 3 h incubation was selected for the following experiments. As a positive control, washed platelets were incubated with thrombin, which was found to significantly induce ROS production (Figure 1B).

Several potential sources of ROS have been suggested, including NADPH oxidase and the mitochondrial respiratory chain [15]. NADPH oxidase-derived ROS play an important role in agonist-stimulated platelet activation [32,33]. Moreover, several reports support a role for NADPH oxidase in hyperthermia-induced tumor cell apoptosis [19,20]. In order to investigate the sources of ROS in hyperthermia-treated platelets, we first studied the effect of NADPH oxidase in hyperthermia-induced ROS production. We used the NADPH oxidase inhibitors DPI and apocynin. We found that DPI partially inhibited ROS generation in hyperthermia-treated platelets, whereas apocynin did not (Figure 1C), suggesting that NADPH oxidase might play an insignificant role in hyperthermia-induced ROS production.

The electron transport chain of the mitochondria is another major source of cellular ROS, particularly in the context of complexes I, II and III [15,16]. We used the mitochondria-targeted ROS antagonist Mito-TEMPO, and the general ROS antagonist N-acetylcysteine (NAC) to ascertain whether mitochondria were a major source of ROS in hyperthermia-treated platelets (Figure 1D). We found that Mito-TEMPO significantly inhibited hyperthermia-induced ROS generation as compared with the solvent control, and treatment with NAC only partially inhibited ROS production. These data demonstrate that mitochondria are a major source of ROS in hyperthermia-treated platelets.

Several studies have shown that nitric oxide synthase (NOS), cyclooxygenase (COX) and lipoxygenase (LOX) are involved in ROS production in agonist-stimulated platelets [33,34]. To demonstrate whether these enzymes are involved in hyperthermia-induced ROS production, ETYA, which is an inhibitor of both COX and LOX, and L-NAME, which is an inhibitor of NOS, were used. The data indicate that these enzymes inhibitors did not block hyperthermia-induced ROS production in platelets (Figure 1E), which suggested that NOS, COX and LOX might play an insignificant role in hyperthermia-induced ROS production.

### Hyperthermia increases mitochondrial superoxide production

**Figure 2:**
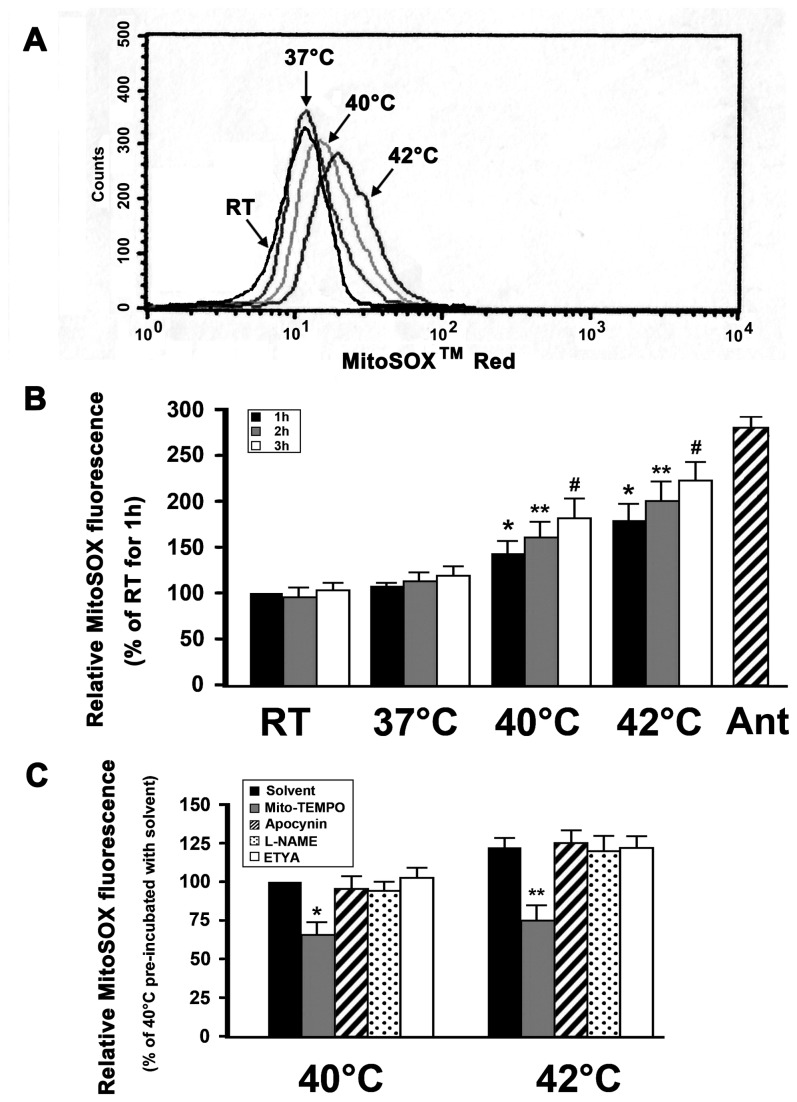
Hyperthermia increase mitochondrial superoxide production. (A and B) MitoSOX^TM^ Red-loaded platelets were incubated at the indicated temperatures for 3 h (A) or1-3 h (B). As a positive control, loaded platelets were incubated with antimycin A at 37°C for 30 min. Samples were then analyzed by flow cytometry. Representative flow cytometric histogram is shown (A). Data are expressed as a percentage of platelets which were incubated at RT for 1h (B). Percentage of RT 1h is presented as mean ± SEM from three independent experiments. *P<0.017 (after a Bonferroni correction) compared with RT 1 h, **P<0.017 (after Bonferroni correction) as compared with RT 2 h, #*P*<0.017 (after Bonferroni correction) as compared with RT 3 h. (C) MitoSOX^TM^ Red-loaded platelets were pre-incubated with apocynin, Mito-TEMPO, L-NAME, ETYA, or solvent control at 37°C for 15 min, and then incubated for 3 h at 40°C or 42°C, and further analyzed by flow cytometry. Data are expressed as a percentage of platelets that were pre-incubated with solvent control at 37°C for 15 min and then incubated at 40°C for 3 h. Percentage of 40°C platelets pre-incubated with solvent control is presented as mean ± SEM from three independent experiments. *P<0.013 (after Bonferroni correction) as compared with solvent control at 40°C, **P<0.013 (after Bonferroni correction) as compared with solvent control at 42°C. Antimycin A is labeled as Ant.

To assist in confirming that mitochondria were a major site of ROS production in hyperthermia-treated platelets, we used MitoSOX^TM^ Red fluorescence, which detects superoxide synthesis, to quantify mitochondrial ROS (Figure 2A and 2B). We found that mitochondrial superoxide production was increased with increasing temperature and in a time-dependent manner. In addition, Mito-TEMPO significantly inhibited hyperthermia-induced mitochondrial ROS generation as compared with the solvent control (Figure 2C). By contrast, inhibitors of NADPH oxidase, NOS, COX and LOX did not (Figure 2C). Together, these observations further confirm that hyperthermia might increase mitochondrial ROS production in platelets. As a positive control, washed platelets were incubated with antimycin A, which is known to increase mitochondrial ROS production [35]. We found that antimycin A markedly induced mitochondrial ROS production in platelets (Figure 2B).

### Hyperthermia increases MDA production and cardiolipin peroxidation in platelets

**Figure 3:**
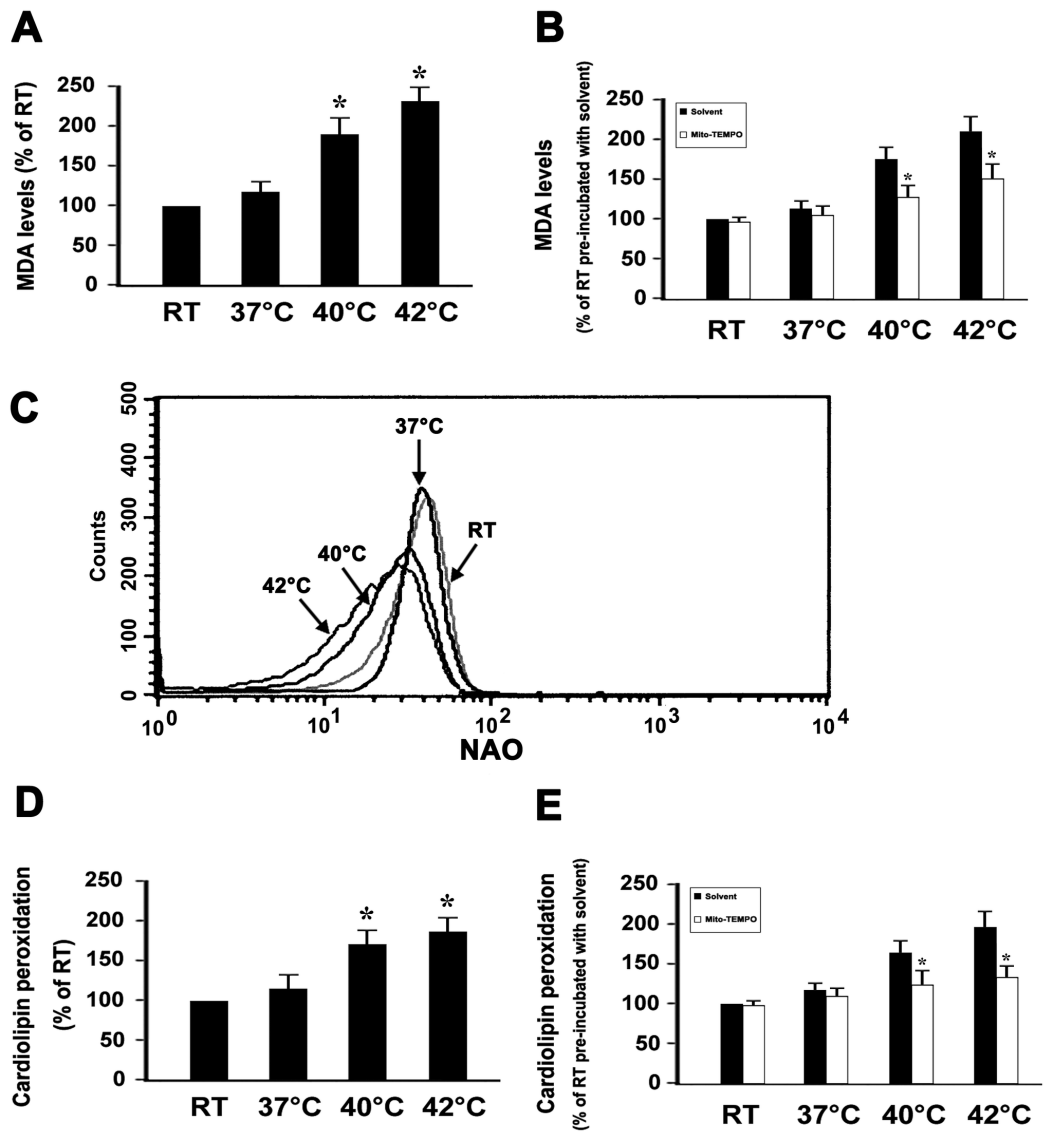
Effect of Mito-TEMPO on the levels of MDA and cardiolipin peoxidation in hyperthermia-treated platelets. (A and B) Washed platelets were incubated without (A), or with (B) Mito-TEMPO and solvent control at 37°C for 15 min, and then incubated at different temperatures for 3 h. MDA levels were detected as described in Methods. (C–E) Washed platelets were incubated without (C and D), or with (E) Mito-TEMPO and solvent control at 37°C for 15 min, and then incubated at different temperatures for 3 h. Cardiolipin peoxidation was detected as described in Methods. Representative flow cytometric histogram is shown (C). (A and D) Data are expressed as a percentage of platelets which were incubated at RT for 3h. Percentage of RT is presented as mean ± SEM from three independent experiments. *P<0.017 (after Bonferroni correction) as compared with RT. (B and E) Data are expressed as a percentage of platelets that were pre-incubated with solvent control at 37°C for 15 min and then incubated at RT for 3 h. Percentage of RT platelets pre-incubated with solvent control is presented as mean ± SEM from three independent experiments. *P<0.05 as compared with solvent control at an identical temperature.

Phospholipids are rich in unsaturated fatty acids that are particularly susceptible to ROS attack, which promotes lipid peroxidation. Several lines of evidence have shown that cellular apoptosis caused by hyperthermia is due to oxidative stress with subsequent lipid peroxidation [36,37]. In order to demonstrate whether hyperthermia induces lipid peroxidation in platelets, we detected the production of MDA, which is a sensitive indicator of ROS-mediated lipid peroxidation [28]. Generation of MDA was increased in a temperature-dependent manner, suggesting that hyperthermia induces platelet lipid peroxidation (Figure 3A). To investigate whether mitochondria-derived ROS were involved in hyperthermia-induced lipid peroxidation, platelets were pre-incubated with Mito-TEMPO or with the solvent control. We showed that hyperthermia-induced MDA production was inhibited by Mito-TEMPO (Figure 3B), indicating that hyperthermia-induced MDA production is mediated by mitochondrial ROS.

Mitochondria are the primary site of ROS production and a target for oxidative stress [14]. In order to obtain more information with regard the deleterious effects of hyperthermia-induced mitochondrial ROS on platelets, we next assessed the effects of hyperthermia on cardiolipin, which is an inner membrane phospholipid of mitochondria. Cardiolipins contain polyunsaturated fatty acid residues, and are thus highly prone to oxidation [38]. Oxygenated cardiolipin appears to be essential for mitochondrial membrane permeabilization and release of pro-apoptotic factors into the cytosol [38,39]. Our above observations indicated that mitochondria are the major source of ROS in hyperthermia-treated platelets. To further explore whether hyperthermia induced cardiolipin peroxidation, we used the fluorescent dye NAO to estimate cardiolipin peroxidation. NAO binds to cardiolipin with high affinity, and the fluorochrome loses its affinity for peroxidized cardiolipin [27]. We found that cardiolipin peroxidation was increased in a temperature-dependent manner (Figure 3C and 3D). To determine if mitochondria-derived ROS was involved in hyperthermia-induced cardiopin peroxidation, platelets were pre-incubated with Mito-TEMPO or solvent control before the treatment with hyperthermia. Our observations were that hyperthermia-induced cardiolipin peroxidation was inhibited by Mito-TEMPO (Figure 3E), indicating that hyperthermia-induced cardiolipin peroxidation is mediated by mitochondrial ROS.

### Mito-TEMPO inhibited hyperthermia-induced platelet apoptosis

**Figure 4:**
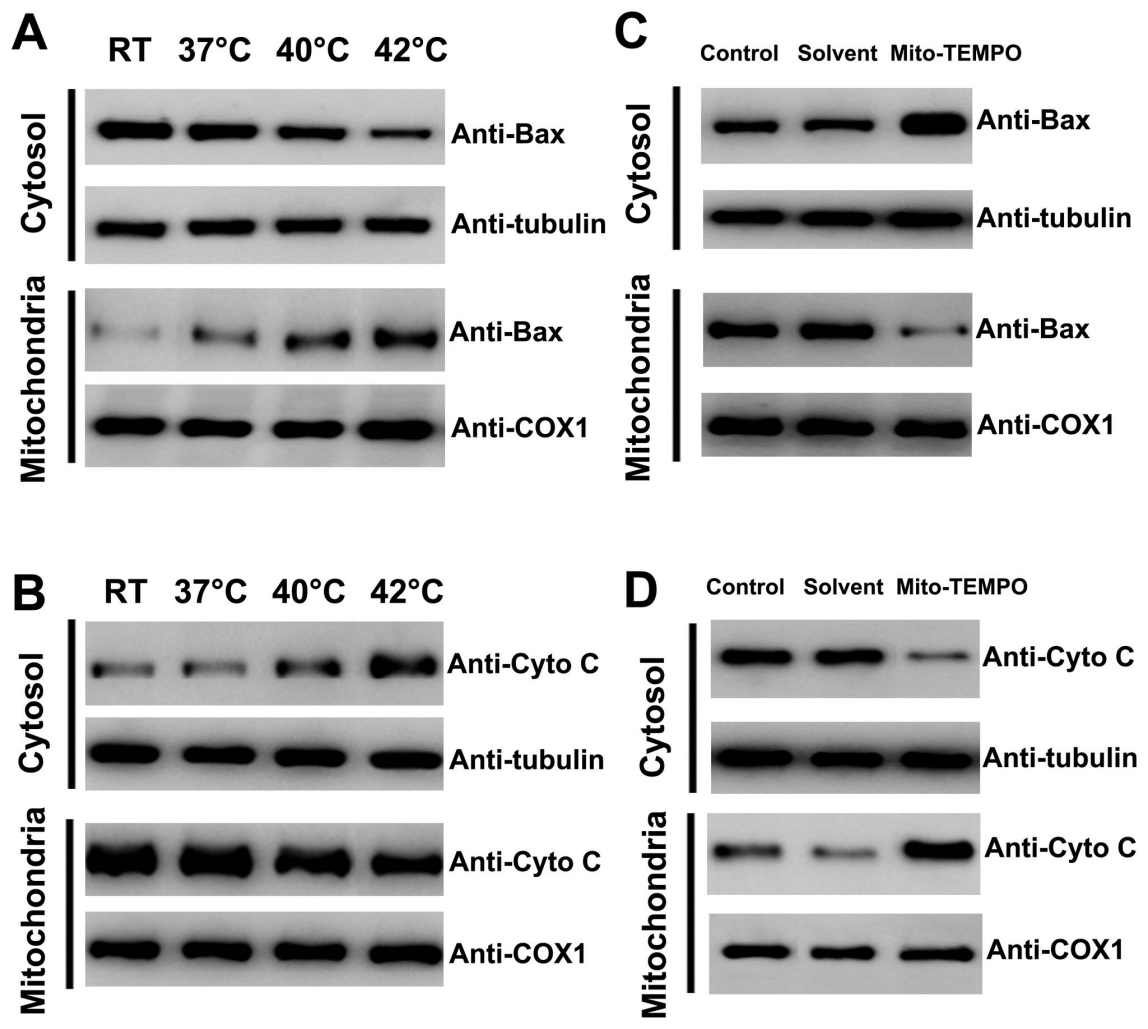
Effect of Mito-TEMPO on Bax mitochondrial translocation and cytochrome C release in hyperthermia-treated platelets. (A–D) Washed platelets were incubated at different temperatures for 3 h (A and B), or pre-incubated with Mito-TEMPO and solvent control at 37°C for 15 min and then incubated at 42°C for 3 h (C and D). Treated platelets were lysed, and cytosol and mitochondrial fractions were isolated and analyzed by Western blot with anti-Bax (A and C), and anti-cytochrome C (B and D). Cytochrome C oxidase subunit 1 (COX1) and tubulin were used as internal controls. Representative data of three independent experiments are presented. Cytochrome C is labeled as Cyto C.

Bax translocation to the mitochondria is a key event that regulates the release of proteins like cytochrome C and Smac/Diablo from the mitochondria, which leads to downstream apoptotic events [40]. Thus, to further explore whether hyperthermia could induce mitochondrial translocation of Bax and cytochrome C release, platelets were pretreated across a range of temperatures and subjected to isolation and analysis of mitochondrial and cytosolic fractions (Figure 4A and 4B). We found that hyperthermia significantly promoted mitochondrial translocation of Bax and cytochrome C release. In order to determine whether Mito-TEMPO affected mitochondrial translocation of Bax and cytochrome C release in hyperthermia-treated platelets, washed platelets were pre-incubated with Mito-TEMPO or solvent control before hyperthermia treatment (Figure 4C and 4D). We showed that Mito-TEMPO significantly inhibited hyperthermia-induced mitochondrial translocation of Bax and cytochrome C release. These observations suggested that mitochondrial ROS play pivotal roles in hyperthermia-induced mitochondrial translocation of Bax and cytochrome C release.

**Figure 5:**
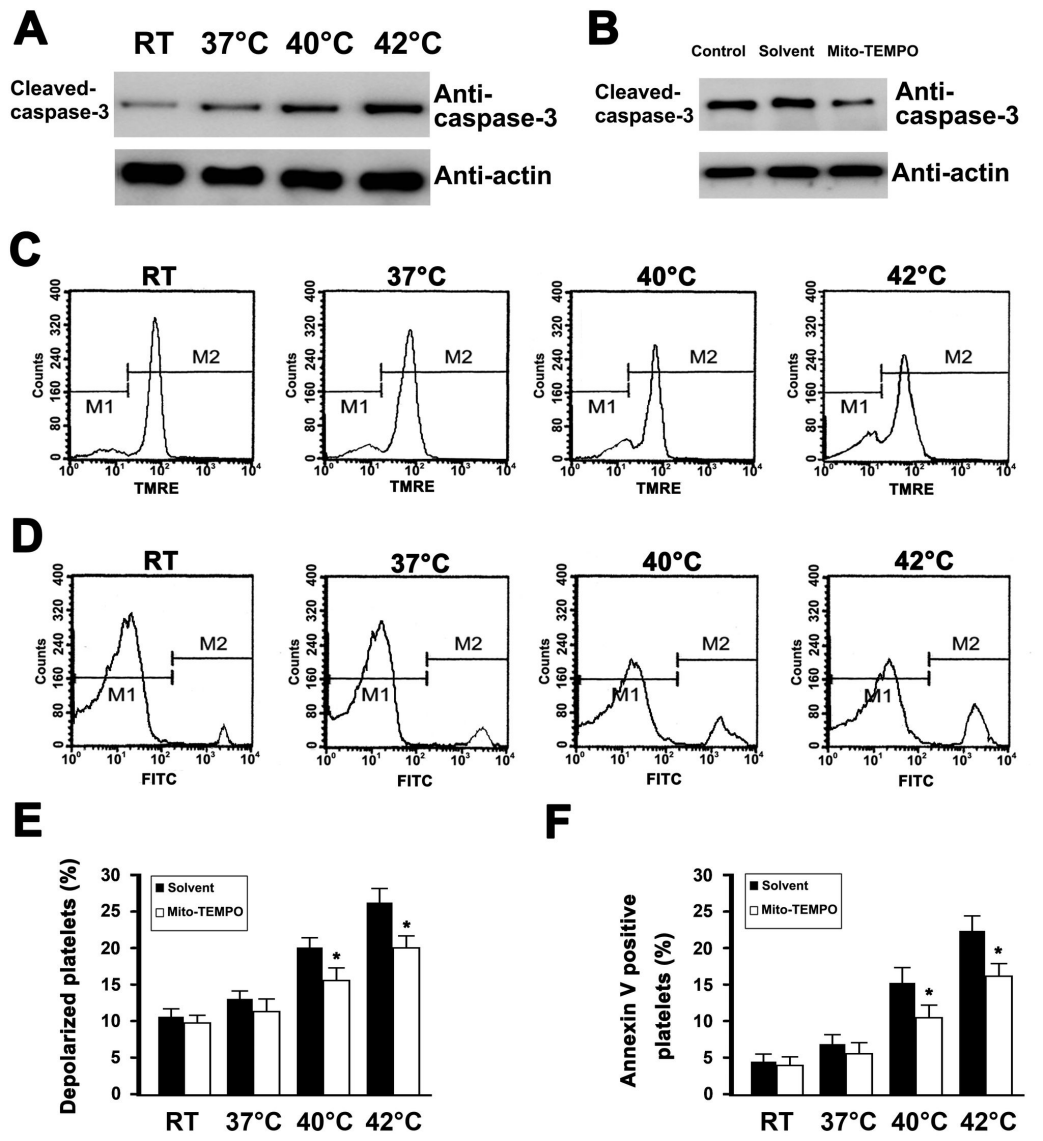
Effect of Mito-TEMPO on caspase-3 activation, depolarization of ΔΨm, and PS exposure in hyperthermia-treated platelets. (A and B) Washed platelets were incubated at indicated temperatures for 3 h (A) or pre-incubated with Mito-TEMPO and solvent control at 37°C for 15 min and then incubated at 42°C for 3 h (B). Treated platelets were lysed and analyzed by Western blot with anti-cleaved p17 fragment of caspase-3. Actin levels were assayed to demonstrate equal protein loading. Representative results of three independent experiments are presented. (C–F) Washed platelets were pre-incubated without (C and D) or with Mito-TEMPO and solvent control at 37°C for 15 min (E and F), and then incubated at the indicated temperature for 3 h. Treated platelets were incubated with TMRE (C and E), or annexin V-FITC (D and F), and analyzed by flow cytometry. Representative flow cytometric histograms are shown (C and D). Data are expressed as a percentage of platelets that were pre-incubated with solvent control at 37°C for 15 min and then incubated at RT for 3 h (E and F). The percentage of RT platelets pre-incubated with solvent is presented as mean ± SEM from three independent experiments. *P<0.05 as compared with solvent control at an identical temperature.

In previous studies, we have reported hyperthermia-induced apoptosis in platelets, including depolarization of ΔΨm, caspase-3 activation and PS exposure [6]. To investigate whether mitochondria-derived ROS were involved in hyperthermia-induced platelet apoptotic events, Mito-TEMPO was pre-incubated with platelets before to hyperthermia treatment (Figure 5). We found that Mito-TEMPO significantly inhibited hyperthermia-induced ΔΨm dissipation, caspase-3 activation, and PS exposure. Together, these data indicate that mitochondrial-derived ROS play a pivotal role in hyperthermia-induced platelet apoptosis.

### Mito-TEMPO ameliorates hyperthermia-impaired platelet function

**Figure 6:**
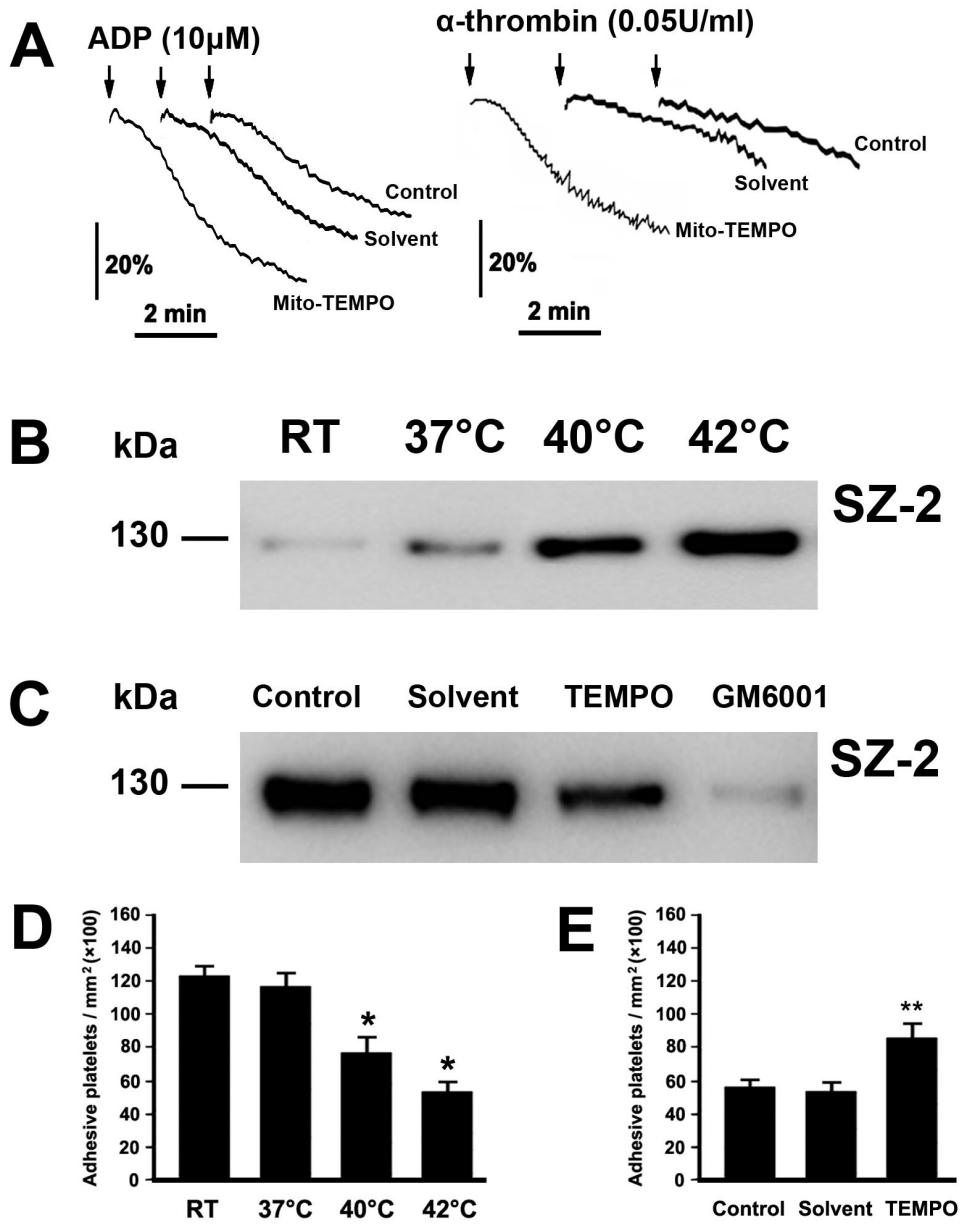
Mito-TEMPO ameliorates hyperthermia-impaired platelet function. (A) Platelet aggregation was performed as described in Methods. Representative traces from three independent experiments are shown. (B and C) Washed platelets were incubated at the indicated temperatures for 3 h (B), or pre-incubated with Mito-TEMPO, GM6001 and solvent control at 37°C for 15 min, followed by incubation at 42°C for 3 h (C). Western blot was performed as described in Methods. (D and E) Washed platelets were incubated at indicated temperatures for 3 h (D), or pre-incubated without (control) or with Mito-TEMPO and solvent at 37°C for 15 min, and then incubated at 42°C for 3 h (E). Platelet adhesion was performed as described in Methods. The results shown are the mean ± SEM of cell number/mm^2^. *P<0.017 (after Bonferroni correction) as compared with RT, **P<0.025 (after Bonferroni correction) as compared with control. Mito-TEMPO is labeled as TEMPO.

Platelets play a central role in maintaining the integrity of the endothelium and biological hemostasis. Our previous work found that hyperthermia impaired ADP- and α-thrombin-induced platelet aggregation [6]. In order to explore whether mitochondria-derived ROS played a role in hyperthermia-impaired platelet aggregation, washed platelets were pre-incubated with Mito-TEMPO or the solvent control before hyperthermia treatment (Figure 6A). We found that Mito-TEMPO significantly inhibited the decrease in ADP- and thrombin-induced platelet aggregation.

Willems et al. reported that the effect of phorbol 12-myristate-13-acetate (PMA) on ADAM17 activity in HeLa cells was inhibited by the mitochondrial electron transport chain inhibitor MitoQ, which indicated that mitochondrial ROS regulated activation of ADAM17 in PMA-treated HeLa cells [41]. Our previous work showed that hyperthermia induces ADAM17-mediated GPIbα ectodomain shedding [6]. To verify whether mitochondrial ROS were involved in hyperthermia-induced GPIbα ectodomain shedding, platelets were pre-incubated with/without Mito-TEMPO before hyperthermia treatment (Figure 6B and 6C). We found that glycocalicin, which is a cleaved production of GPIbα [26], gradually increased with increasing temperature (Figure 6B). We also found that mitochondria-targeted ROS antagonist significantly inhibited hyperthermia-induced glycocalicin generation (Figure 6C), suggesting that mitochondrial ROS play a role in hyperthermia-induced GPIbα ectodomain shedding. As a positive control, the ADAM17 inhibitor GM6001 completely blocked hyperthermia-induced the shedding of the GPIbα ectodomain (Figure 6C).

The interaction of GPIbα with vWF at sites of injured blood vessel walls initiates platelet adhesion under flow conditions [26,31]. To test whether hyperthermia-induced GPIbα shedding inhibits GPIbα-dependent platelet function, washed platelets were treated with different temperatures, and then passed through a vWF coated glass capillary at a specific shear rate. Compared with RT, hyperthermia-treated platelets displayed a significant decrease in adhering on the vWF surface (Figure 6D). The observations in Figure 6C indicate that the mitochondria-targeted ROS antagonist significantly inhibited hyperthermia-induced GPIbα ectodomain shedding. To further confirm whether the mitochondria-targeted ROS antagonist improves hyperthermia-impaired platelet adhesion, platelets were pre-incubated with Mito-TEMPO before hyperthermia treatment (Figure 6E). We showed that Mito-TEMPO significantly inhibited hyperthermia-impaired platelet adhesion.

### Hyperthermia decreased MnSOD protein levels and enzyme activity

**Figure 7:**
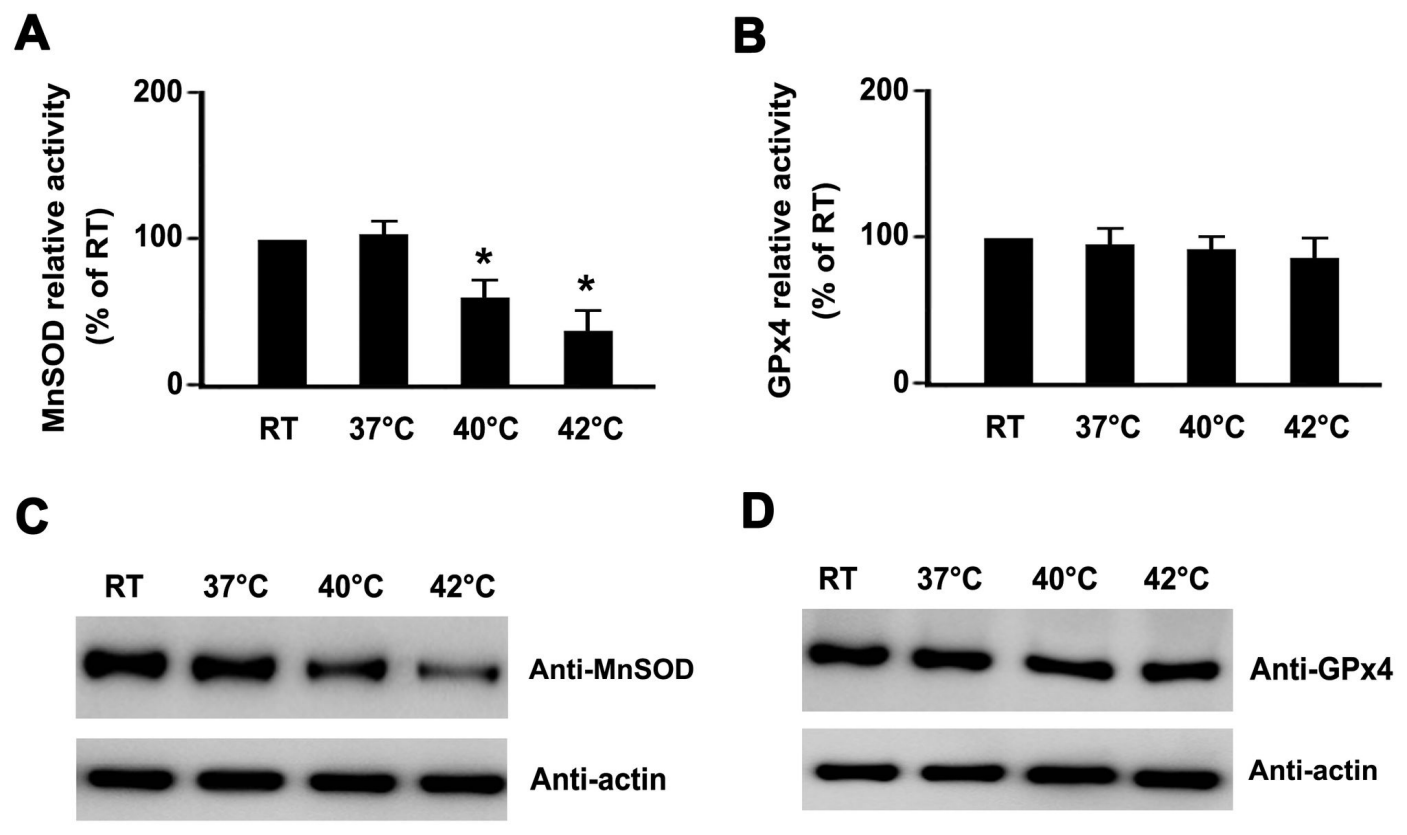
Hyperthermia decreases MnSOD protein levels and enzyme activity. (A and B) Washed platelets were incubated at the indicated temperatures for 3 h. Treated platelets were lysed, and ManSOD activity was measured using a commercially available SOD (A), or GPx4 (B) assay kits. *P<0.017 (after Bonferroni correction) as compared with RT. (C and D) Washed platelets were incubated at the indicated temperatures for 3 h. Treated platelets were lysed and analyzed by Western blot with anti-MnSOD (C), or anti-GPx4 (D). Actin levels demonstrated equal protein loading. Representative results of three independent experiments are presented.

The above observations confirmed that hyperthermia treatment enhanced mitochondrial ROS levels in platelets, and further demonstrated that mitochondrial ROS played a key role in hyperthermia-induced platelet apoptosis. Next, we attempted to determine the possible reasons for enhanced mitochondrial ROS. It was previously shown that both MnSOD and GPx4 play key roles in scavenging mitochondrial ROS [11,12,14]. We hypothesized that hyperthermia reduced the activities of MnSOD and GPx4, and consequently stimulated an increase in mitochondrial ROS. In order to demonstrate our hypothesis, we first quantified the activity of MnSOD and GPx4 using commercially available kits. We found that MnSOD activity was clearly decreased in hyperthermia-treated platelets (Figure 7A), and that GPx4 activity was only slightly reduced (Figure 7B). Next, we explored whether the decrease in MnSOD activity was due to reduced expression of MnSOD protein levels. We found that MnSOD protein levels were markedly decreased in hyperthermia-treated platelets (Figure 7C). However, hyperthermia minimally affected GPx4 protein levels (Figure 7D).

## Discussion

In this report, our findings confirmed that mitochondria were the major sources of hyperthermia-induced ROS generation in platelets. In addition, we demonstrated that mitochondrial ROS play a key role in hyperthermia-induced platelet apoptosis. We also showed that mitochondria-targeted ROS antagonists can inhibit hyperthermia-induced apoptotic events, including cardiolipin peroxidation, mitochondrial translocation of Bax, cytochrome C release, caspase-3 activation, PS exposure and ΔΨm depolarization. Decreased MnSOD protein levels and activity could play an important role in hyperthermia-induced increases in mitochondrial ROS.

Hyperthermia induces ROS generation and apoptosis in various cell types [7,8], and the identities of the cellular sources of ROS remain controversial. We found that mitochondria are the primary source of ROS in hyperthermia-treated platelets based our observations that (1) the mitochondria-targeted ROS scavenger Mito-TEMPO inhibited hyperthermia-induced ROS production, and (2) hyperthermia-induced ROS was detected by the mitochondrial ROS probe MitoSOX^TM^ Red. This finding was consistent with the established role of mitochondria as key sources of ROS generation through the electron transport chain [11]. Several lines of evidence have indicated that heat stress induces ROS production in various cell types [7,8,19,20,25].

However, several studies have shown that NADPH oxidase is a major source of ROS in hyperthermia-treated cells [19,20]. Moon et al. recently reported that hyperthermia augments NADPH oxidase 1 (NOX1) mRNA expression, and leads to enhanced NADPH oxidase activity [19]. Platelets do not have nuclei and thus do not have the ability to transcriptionally regulate protein expression [42]. In addition, many studies have used DPI as an NADPH oxidase inhibitor. Lambert et al. reported that DPI acutely inhibits ROS production by the mitochondrial complex I [43]. Our observations also confirm that DPI partially inhibited hyperthermia-induced ROS production. By contrast, the NADPH oxidase inhibitor apocynin did not. Therefore, different sources of hyperthermia-induced ROS generation are likely to be dependent on both the cell type and use of pharmacological inhibitors. 

The precise mechanisms responsible for how hyperthermia causes increased levels of mitochondrial ROS remain undetermined. The reasons may be manifold. On the one hand, hyperthermia might increase mitochondrial ROS generation. During normal mitochondrial function, a small percentage of electrons from the electron transport chain reduce oxygen to form superoxide. During mitochondrial dysfunction, this leak of electrons is increased. Tissier et al. have previously reported that mild hypothermia preserves mitochondrial function and reduces mitochondrial ROS generation [44]. On the contrary, dysfunction in mitochondrial respiration might increase the formation of ROS in mitochondria [45,46], Swerdlow et al. also reported that enhanced production of mitochondrial ROS was linked to mitochondrial dysfunction [47]. It has also been reported that hyperthermia could induce mitochondrial dysfunction [48] and thus augment mitochondrial ROS production. On the other hand, hyperthermia might provoke decreased antioxidant capacity in mitochondria. Our studies found that hyperthermia caused a decrease of MnSOD activity in platelets. This observation might partly explain the accumulation of mitochondrial ROS in hyperthermia-treated platelets.

In addition, it remains largely unknown how hyperthermia provoked a decrease in MnSOD activity. MnSOD activity was likely regulated by several pathways; 1) MnSOD protein levels were lowered due to decreased synthesis or increased degradation, 2) the activity of MnSOD was reduced although MnSOD protein levels were unaltered. Hyperthermia can drive the ubiquitination of myeloid cell leukemia-1 (Mcl-1), and induce Mcl-1 degradation [49]. Whether hyperthermia could induce MnSOD, which could then subsequently promote ubiquitination of proteins and their degradation remains to be further studied.

The functional role of mitochondrial ROS in hyperthermia-induced platelet apoptosis was determined by pre-treating platelets with a mitochondria-targeted ROS scavenger before hyperthermia treatment and then analyzing apoptotic markers. The mitochondrial-targeted ROS scavenger was found to be effective in inhibiting hyperthermia-induced platelet apoptosis. These observations are in good agreement with what is currently known about the synthesis of ROS in biological systems, and indicates that mitochondrial ROS are key mediators of hyperthermia-induced platelet apoptosis. However, the question remains of how mitochondrial ROS trigger platelet apoptosis? It has been previously reported that mitochondria-derived ROS plays a pivotal role in triggering apoptosis in various cell types [14,23,24]. It has been reported that mitochondria-derived superoxide anions play a pro-apoptotic role by causing the down-regulation and degradation of the Bcl-2 protein in an ubiquitin proteasomal-dependent pathway [50].

Bcl-2 is a key regulator of the intrinsic pathway by interfering with cytochrome C release through its interaction with Bax [51]. Other studies have shown that mitochondrial ROS easily oxidized cardiolipin, and oxidized cardiolipin appears to be essential for mitochondrial membrane permeabilization and release of pro-apoptotic factors into the cytosol [52]. Conversely, prevention of cardiolipin peroxidation leads to inhibition of apoptosis [53]. These findings suggest that cardiolipin might be a crucial molecule that regulates the initiation of apoptosis. Our data demonstrate that hyperthermia increased cardiolipin peroxidation and that mitochondrial ROS plays an important role in hyperthermia-induced cardiolipin peroxidation. Future studies will investigate whether cardiolipin peroxidation plays an important role in initiating hyperthermia-induced platelet apoptosis.

In conclusion, our study provides direct evidence that hyperthermia increases mitochondria-derived ROS production in platelets, which in turn, induces platelet apoptosis and GPIbα ectodomain shedding. Hyperthermia induces platelet apoptosis, which in turn impairs platelet function and decreases the numbers of peripheral platelets; an outcome that manifest as increased hemorrhage or thrombocytopenia. Recently, Zhang et al. reported that P2Y12 activation protects platelets from apoptosis [54]. Application of P2Y12 activation in combination with hyperthermic therapy might be a physiologically important option to counter hyperthermia-induced platelets apoptosis.
